# Personal and situational determinants of perception of important life events

**DOI:** 10.1192/j.eurpsy.2023.795

**Published:** 2023-07-19

**Authors:** V. Umlauft

**Affiliations:** Social psychology, Lomonosov Moscow State University, Vienna, Austria

## Abstract

**Introduction:**

The present time is characterized by the instability of society, the authoritarian state, social insecurity, and the events of the global pandemic. One of the main properties of modernity is the continuous nature of the social changes taking place in it. Social uncertainty, instability and social insecurity complicate the usual strategy for constructing ideas about the world around us. In connection with certain factors, events in a person’s life can be defined as significant.

**Objectives:**

The purpose of this study was to ckeck the fact that important life events are something associated with stress and have a negative emotional connotation.

**Methods:**

The methodological basis of the research was the qualitative study: semi-structured interviews, including projective techniques (case and content analysis) were carried out. N = 50 residents of Russia and Austria, age 16-65.

**Results:**

The hypothesis about important life events was completely confirmed. It turned out that important life events were described by the respondents mainly as negative, that is, in most cases (more than 70% of the total number of answers), important events were associated as something associated with stress, having a negative emotional connotation. It is interesting that mentions of joyful and happy events were much less frequent than negative ones, while there were practically no descriptions of the experience of vivid emotions accompanying any long-awaited events. At the same time, important events are quite extensively associated with stress and critical emotional experiences, often with mental problems: “shock”, “crisis”, “debts”, “severe depression”, “complete collapse”, “everything goes to hell”.

**Conclusions:**

Important life events for a person are rather negatively assessed events, as well as events associated with stress, rather than positively assessed events.

**Disclosure of Interest:**

None Declared

